# Post-Traumatic Stress Symptoms in Parents of Preschoolers First Diagnosed with Autism: Gender Differences and Correlations with Broad Autism Phenotypes

**DOI:** 10.3390/ijerph22111642

**Published:** 2025-10-28

**Authors:** Claudia Carmassi, Valerio Dell’Oste, Eugenia Conti, Sara Fantasia, Andrea Bordacchini, Berenice Rimoldi, Virginia Pedrinelli, Lorenzo Conti, Roberta Battini, Sara Calderoni

**Affiliations:** 1 Department of Clinical and Experimental Medicine, University of Pisa, 56126 Pisa, Italy; valerio.delloste@gmail.com (V.D.); eugenia.conti@unipi.it (E.C.); dr.fantasiasara@gmail.com (S.F.); andreabordacchini@gmail.com (A.B.); bererimo@gmail.com (B.R.); virginiapedrinelli@gmail.com (V.P.); lorenzo.conti.terp@gmail.com (L.C.); roberta.battini@unipi.it (R.B.); sara.calderoni@unipi.it (S.C.); 2 Department of Mental Health and Addiction, Azienda USL Toscana Nord-Ovest, 55100 Lucca, Italy; 3 Department of Developmental Neuroscience, IRCCS Stella Maris Foundation, 56128 Pisa, Italy; 4 Department of Biotechnology, Chemistry and Pharmacy, University of Siena, 53100 Siena, Italy; 5 Department of Mental Health and Addiction, Azienda USL Toscana Nord-Ovest, 57100 Livorno, Italy

**Keywords:** post-traumatic stress symptoms, autism spectrum disorder, ASD, autistic traits, global functioning, parents, subthreshold autism spectrum

## Abstract

(1) Background: A child’s new diagnosis of autism can represent a highly stressful event for parents. Subthreshold autistic traits (ATs) have been linked to higher vulnerability to psychopathology when exposed to stressful situations, and high rates of ATs have been reported among parents of autistic children. This study aimed to evaluate post-traumatic stress spectrum symptoms (PTSS) in parents of preschool children newly diagnosed with autism and to explore differences between mothers and fathers, besides the correlations with ATs. (2) Methods: A total of 134 parents of children newly diagnosed with autism were assessed by trained psychiatrists from the University of Pisa using the Autism Spectrum Quotient (AQ), the Adult Autism Subthreshold Spectrum-Self Report (AdAS-SR), the Trauma and Loss Spectrum-Self Report (TALS-SR), and the Social and Occupational Functioning Assessment Scale (SOFAS). (3) Results: Approximately 10% of parents met DSM-5-TR criteria for symptomatologic PTSD, with nearly 40% experiencing partial PTSD symptoms related to their child’s diagnosis. Mothers showed higher PTSD rates than fathers. The ATs significantly correlated with elevated TALS-SR scores, and logistic regression revealed a positive association between ATs and PTSD (*p* < 0.001). Linear regression analysis indicated that higher TALS-SR scores predicted lower SOFAS scores (*p* = 0.004). (4) Conclusions: These findings highlight the potential traumatic impact of a child’s new autism diagnosis on parents, particularly mothers and individuals with ATs. The results underscore the importance of targeted support strategies for parents, considering their key role in early interventions. Further research is needed to better understand parental psychological responses and to enhance support systems, ultimately improving family wellbeing and child outcomes.

## 1. Introduction

Receiving a diagnosis of severe illness in one’s child can represent a highly stressful situation for even the most resilient parents. This is not only because of the fear of threat to the child’s life but also for that on their future outcomes, the need to cope with the daily circumstances related to the illness, the medical procedures, and the continuous treatments needed [1,2,3,4]. Strong emotional reactions about the child’s illness, including feelings of fear, anxiety, powerlessness, and anger, have been widely described. While the emotional reactions of parents receiving a medical diagnosis for their child can vary from case to case, they follow a typical course: initially, a state of shock prevails, followed by disbelief that can alternate with anguish, sadness, helplessness, and anger. These are normal adaptation reactions, and they appropriately occur and manifest openly [5,6]. However, for some families, the medical experience may represent a significant stressful event.

Among psychopathological reactions to stressful or traumatic events, Post-Traumatic Stress Disorder (PTSD) [7] and Post-Traumatic Stress Spectrum symptoms (PTSS) [8] are the most relevant. The DSM-5-TR currently considers life-threatening illnesses or debilitating medical conditions potentially related to PTSD to be limited to sudden medical emergencies. According to DSM-5-TR criteria, in fact, “a medical catastrophe concerning one’s child” was added in the list of events that can lead to PTSD, specifying that the event must represent a condition that immediately endangers life [7]. However, an increasing number of studies have shown that a diagnosis has an impact even when it does not pose an immediate threat to a child’s life [9,10,11,12]. From this perspective, PTSD has deservedly received more attention among parents and caregivers of pediatric patients affected by serious but different conditions such as cancer, leukemia/lymphoma, diabetes, acute (ischemic, hemorrhagic, traumatic) or chronic (epilepsy) neurological disorders, with studies pointing out the potentially traumatic role of such experiences [13,14,15,16,17]. Other studies have explored medical conditions such as traumatic injuries, pediatric ICU admissions, cardiac diseases, or epilepsy [18,19,20,21]. In this context, a meta-analysis by Wilcoxon and colleagues [18] suggested a PTSD prevalence rate of between 17% and 14% in parents of children who had experienced a road accident, physical trauma, or admission to a pediatric or neonatal intensive care unit. Similar results emerged from studies conducted on parents of children affected by cardiac diseases [18], type 1 diabetes, cancer [9,10,11,12,13,14,15,16,17,18,19,20], and severe epilepsy [21].

One of the most complex diagnoses that a parent may have to face is Autism. Autism is recognized as an early-onset neurodevelopmental disorder, in which genetic predisposition plays a major role, often combined with de novo variations and their interplay with environmental factors. Such mechanisms seem to alter neural connectivity, producing a cascade of effects that compromise cognitive processes [22,23,24,25]. Clinically, it is characterized by significant impairments in social communication and interaction, accompanied by repetitive patterns of behavior and restricted interests, with intellectual disability present in some but not all cases [7]. Specific symptoms include difficulties with social-emotional reciprocity as well as in verbal and nonverbal communication, developing relationships, besides unusual sensory reactions to stimuli, repetitive motor movements, rigid adherence to routines, and highly focused interests. This leads to a disorder with a possible significant impact on everyday life that affects the whole family [26]. Firth and Dryer (2013) [27] reported that children’s behavioral and emotional impairments predicted parents’ overall levels of distress (i.e., stress/tension, anxiety, and depression), but not the stress associated with parenting, as well as the child’s social impairment severity predicted parenting-specific stress. Studies conducted on parents of recently diagnosed children demonstrated how autism symptoms have a direct correlation with depressed mood and post-traumatic stress symptoms, with a notable correlation to the severity of the child’s problematic behaviors [28]. Stress proliferation must also be considered, as families face the combination of autism symptoms and enhanced caring needs, so that other key areas of their life, such as work, physical health, relationship with a spouse or with other family members, may be impaired [29,30].

Several risk factors related to PTSD development in parents have been reported, such as female gender, acute stress disorder life situation, predisposition, anxiety and depression, and negative coping style [31,32]. In particular, mothers and fathers seem to be differently affected by parental stress after the diagnosis. Davisa and Carted noted how regulatory problems were mainly associated with maternal stress, and externalizing behaviors were of major concern for fathers, while delays in social relatedness were associated with distress in both parents [33]. Mothers seem to be majorly impacted, as they tend to score higher on depression, anxiety, and stress scales, highlighting female gender as a factor associated with higher stress levels [32]. Interestingly, as with other traumatic events, some authors pointed out how circumstances regarding the moment of the diagnosis can also lead to different health outcomes in parents, and differences in perceived quality of information and time needed to complete the diagnostic process have been linked to notable variances in parenting distress [34,35].

There is evidence that autistic traits (ATs) may represent vulnerability factors to the traumatic impact of events [36,37]. In this perspective, the communication of the diagnosis of autism in a child may entail a threatening impact on their parents [38]. A growing body of research has highlighted that first-degree relatives of individuals with autism often exhibit a range of subclinical manifestations, giving rise to the concept of the “broader autism phenotype” (BAP). This term refers to the presence of autistic traits below the diagnostic threshold that can be identified in close relatives, especially in parents, both at the clinical and neurobiological level [39,40]. Evidence suggests that BAP may be linked to adverse mental health outcomes in these parents, including higher levels of stress and depressive symptoms [41]. On the other hand, the autistic traits of parents uniquely influence the interaction between the child and the family. Parents of autistic children might be in the best position to understand their children’s needs [42], but they could simultaneously experience increased stress, depression, and anxiety due to the challenges of raising an autistic child [31,43]. Additionally, these parents may relive through their children the difficulties and traumatic experiences from their own childhood, and face challenges in managing the social relationships necessary for effective parenting [31].

All this mentioned emphasizes the importance of keeping trauma-related psychopathology of caregivers in mind, both for the possible impact on their overall wellbeing and functioning, and for their primary role in their children’s support and treatment. Consistently, it would allow us to address effective strategies of parental support and the implementation of their potential role as caregivers. We hypothesized that a new diagnosis of autism in one’s young child, in preschool age, may have a potentially traumatic impact on parents, with potentially different effects on mothers and fathers. We also hypothesized that the presence of ATs may negatively impact the mental health burden, being related to higher post-traumatic stress symptoms and more severe work and social functioning impairment. Thus, in line with previous studies [44,45], we aimed to evaluate the possible traumatic impact of one’s child being diagnosed with autism and to investigate PTSS in parents of children diagnosed with autism. Moreover, we focused on individual parental couples to disentangle the different impacts on mothers and fathers. The secondary aim of the present study was to detect whether ATs eventually reported by parents may impact the PTSS rates detected. We believe that this study could contribute to better identifying parents majorly in need of support measures, since trauma-related psychopathology, aside with autistic traits in caregivers, could impair their functioning and make it harder for them to accomplish their role in children’s development [30,46]. All of these aspects could be of great interest in the field to empower the treatment of children and their relatives.

## 2. Materials and Methods

This is an observational, cross-sectional study held in a tertiary care University Hospital in Italy, jointly by the Psychiatric Clinic of the University of Pisa (Pisa, Italy) and the IRCCS Stella Maris Foundation (Pisa, Italy). Parents of young preschoolers aged below 6 years with a diagnosis of ASD according to the DSM-5-TR criteria [7], admitted for evaluation at the outpatient IRCCS Stella Maris Foundation, were consecutively recruited for participation. Parental couples were asked to participate in the study and, after signing a written informed consent, were interviewed to detect the potentially traumatic impact of the child’s diagnosis of autism, and they were assessed at the IRCCS Stella Maris Foundation by trained clinicians of the Psychiatric Clinic of the University of Pisa. Parents were excluded if they had insufficient knowledge of the Italian language or other conditions limiting verbal communication. All participants who met the inclusion criteria were invited to sign a written informed consent form after being fully informed about the study procedures and given the chance to ask clarifying questions. The research was carried out in compliance with the principles of the Helsinki Declaration and obtained authorization from the local Ethics Committee.

### 2.1. Instruments and Assessment

The psychiatric evaluation comprised the administration of the Autism Spectrum Quotient (AQ), the Adult Autism Subthreshold Spectrum–Self Report (AdAS-SR), the Trauma and Loss Spectrum–Self Report (TALS-SR), and the Social and Occupational Functioning Assessment Scale (SOFAS).

The AQ [47,48] is a widely adopted self-report instrument designed to evaluate autistic traits in adults without intellectual disabilities. It has demonstrated satisfactory test–retest stability and interrater reliability. The tool consists of 50 items covering five domains: social abilities, attention shifting, attention to detail, communication, and imagination. In its validation, a threshold score above 32 was identified as indicative of clinically relevant autistic features. Scores of 23 or higher have instead been associated with the possible presence of the broader autism phenotype (BAP), reflecting subthreshold expressions of autism such as atypical communication and cognitive styles, intense and persistent interests, and a rigid or socially detached personality [48].

The AdAS Spectrum is an instrument for assessing subthreshold, isolated, or atypical or gender-specific features of the autism spectrum in adults with average intelligence and no language impairment. It consists of 160 items with dichotomous responses (yes/no) divided into 7 domains: (1) symptoms in childhood or adolescence; (2) verbal communication; (3) non-verbal communication; (4) empathy; (5) Inflexibility and adherence to routine; (6) restricted interests and rumination; (7) Hyper– and hyporeactivity to sensory input. A cut-off score of 70 identifies subjects with marked autistic traits, while a cut-off score of 43 identifies the presence of significant autistic traits, with satisfying levels of specificity and sensitivity, as well as a good agreement with the autistic diagnosis according to DSM-5-TR criteria [39].

The TALS-SR [8] is a self-report measure designed to detect typical, atypical, attenuated, and subthreshold manifestations across the post-traumatic stress spectrum, offering a fully dimensional view of individual psychophysiology. It comprises 116 dichotomous (yes/no) items organized into nine domains: (1) loss events; (2) grief reactions; (3) potentially traumatic events; (4) responses to losses or distressing events; (5) re-experiencing; (6) avoidance and numbing; (7) maladaptive coping; (8) arousal; and (9) personal characteristics/risk factors. A total symptomatologic score was computed as the sum of domains 4 through 8. In line with prior work, the presence of PTSD was evaluated using TALS-SR items from domains V–VIII mapped to DSM-5-TR diagnostic criteria. For the purposes of this study, the instrument was contextualized to assess post-traumatic stress symptoms linked to the child’s autism diagnosis in all participants. Criterion-level scores were derived for each DSM-5-TR PTSD criterion (B, C, D, E) by summing endorsed items: B1–B5 (range 0–5), C1–C2 (0–2), D1–D7 (0–7), and E1–E6 (0–6). Consistent with the DSM-5-TR literature, Partial A PTSD and Partial B PTSD classifications were also considered, defined by meeting 3 of 4 or 2 of 4 B–E symptom criteria, respectively [49,50]. The TALS-SR shows excellent agreement with the interview-based SCI-TALS, with intraclass correlation coefficients between 0.934 and 0.994; the SCI-TALS likewise demonstrates adequate internal consistency, with Kuder–Richardson coefficients exceeding the minimum standard of 0.50 across domains.

The SOFAS, included in the DSM-5-TR, is an observational scale frequently applied in research to evaluate social and occupational functioning along a continuum. Scores range from 1 to 100, with higher values reflecting better levels of functioning. The scale requires that impairments be rated only when they are directly attributable to psychiatric or somatic symptoms.

### 2.2. Statistical Analyses

In descriptive statistics, absolute and relative frequencies (n,%) and means and standard deviations (mean ± SD) are reported for categorical and scalar variables, respectively. The AQ, SOFAS, TALS-SR, and AdAS Spectrum mean scores were calculated in the total sample and in the mothers’ and fathers’ subgroups. The rates of endorsement of DSM-5-TR diagnostic criteria were calculated in the total sample and in both the fathers and mothers. Since the variables examined in the study did not have a normal distribution, non-parametric analyses were performed. Particularly, the nonparametric Mann–Whitney test was used to compare mothers and fathers on AdAS, AQ, and SOFAS scores. The same test was used to compare subjects with and without significant autistic traits scores (AdAS cut-off = 42) on TALS scores. Considering the couples of mothers and fathers paired for one’s child, the nonparametric Wilcoxon signed rank test was utilized to explore gender differences on TALS scores, whereas the McNemar test was utilized to compare mothers and fathers on the rates of endorsement of diagnostic criteria. A logistic regression analysis was also performed with a TALS-SR diagnosis of at least partial DSM-5-TR PTSD as the dependent variable, and AdAS total scores and gender were included. Finally, a linear regression analysis was performed with SOFAS total score as the dependent variable and AdAS total score, TALS-SR Symptomatologic total score, and gender as independent variables. A two-tailed *p*-value test < 0.05 was considered statistically significant. All statistical analyses were performed using Statistical Package for the Social Sciences (SPSS) version 29.0.

## 3. Results

The total sample consisted of 124 parents of autistic children, including 62 fathers and 62 mothers, all parental couples of one child, with a mean age of 39.81 ± 5.98 years. Twenty subjects (16.1%) were single, 84 (67.7%) were married or cohabitating, and 20 (16.1%) were separated or divorced. Forty-eight (38.7%) subjects were graduates or post-graduates; eighty-six (69.3%) parents were employed. A total of 41.1% of the subjects (N = 46) reported a previous psychiatric history, and 22.3% (N = 25) reported a current psychopathological disorder.

Comparing mothers and fathers of each child (Wilcoxon signed rank test), statistically significantly higher TALS-SR domains mean scores emerged in mothers with respect to fathers in: loss events (I) (*p* = 0.002), grief reactions (II) (*p* = 0.002), reactions to losses or upsetting events (IV) (*p* = 0.001), re-experiencing (V) (*p* = 0.007), maladaptive copying (VII) (*p* = 0.032), arousal (VIII) (*p* = 0.025), besides the Total Symptomatologic score (IV + V + VI + VII + VIII) (*p* = 0.001) (see Table 1). Furthermore, 13 (10.3%) parents met DSM-5-TR criteria for full-blown symptomatologic PTSD and 49 (38.9%) for partial PTSD; among these latter 23 (18.3%) met three criteria and 26 (20.6%) met two criteria. Consistently, mothers were significantly more likely to meet DSM-5-TR PTSD criterion B (*p* = 0.011), D (*p* = 0.023), and E (*p* = 0.008) than fathers (see Table 1).

A mean total AdAS score of 30.83 ± 20.82 emerged in the total sample (29.90 ± 21.96 among fathers, with respect to 31.26 ± 19.73 among the mothers, z = −0.724, *p* = 0.469). According to the AdAS spectrum questionnaire, 23 (18.4%) parents reported significant autistic traits, with a mean total AdAS score over the threshold of 43, and 5 (4.0%) autistic symptoms, with a mean total AdAS score over 70. In particular, 11 (17.5%) of the fathers reported a mean total AdAS score over 43, and 3 (4.8%) over 70, compared to 12 (19.4%) and 2 (3.2%) of the mothers, respectively (χ^2^ = 0.247, *p* = 0.884) (see Table 2). In all AdAS domains, there were no statistically significant differences between mothers and fathers.

A mean total AQ score of 13.02 ± 5.98 emerged in the total sample (13.53 ± 6.28 among fathers with respect to 12.52 ± 5.67 among mothers, z = −0.910, *p* = 0.363). Statistically significantly higher AQ mean scores emerged among fathers, with respect to mothers, in the Imagination domain only (*p* = 0.017).

As far as the SOFAS is concerned, total mean scores of 83.67 ± 11.35 emerged in the total samples, particularly, 81.77 ± 11.20 among mothers and 85.65 ± 11.23 among fathers (z = −2.211; *p* = 0.027).

When comparing parents with significant AT, based on the AdAS scores, with respect to those without, statistically significantly higher mean TALS-SR scores emerged in all domains but the maladaptive copying, besides statistically significantly lower SOFAS total scores (see Table 3).

A logistic regression analysis performed with a TALS-SR diagnosis of at least partial DSM-5-TR PTSD as the dependent variable, and AdAS total scores and gender as independent variables, showed a statistically significant (*p* < 0.001) role of the AdAS total scores (see Table 4A). A linear regression analysis performed with SOFAS total score as dependent variable and gender, AdAS total score, and TALS-SR Symptomatologic total score as independent variables, showed a statistically significant (*p* = 0.004) of this latter as a predictor of functional impairment (see Table 4B).

## 4. Discussion

To the best of our knowledge, this is the first study exploring the potentially distressing impact of a new diagnosis of autism in one’s child, comparing differences among couples of mothers and fathers of pre-school children, and evaluating the role of Adult Autism Subthreshold Spectrum on post-traumatic stress symptoms. The results of the present study show a diagnosis of symptomatologic PTSD, according to current DSM-5-TR criteria, in about one-tenth of parents of autistic children, with a further almost 40% of parents suffering from a partial form of the condition. Interestingly, according to our hypothesis, the presence of significant ATs showed a negative impact on post-traumatic stress spectrum symptoms, leading to higher TALS-SR scores.

### 4.1. Gender Differences

Our results showed high rates of symptomatologic PTSD (10.3%) among parents of children newly diagnosed with autism, in line with previous literature on parents of children with severe life-threatening acute or chronic disorders, where PTSD rates between 11% and 19.6% were reported [51,52,53]. Further, higher rates of symptomatologic PTSD emerged among mothers with respect to fathers, particularly when considering parental couples of the same child, in line with previous data on parents of children with a diagnosis of epilepsy [21,44]. Statistically significant gender differences also emerged in the PTSD symptoms, where mothers endorsed more intrusion, negative alteration in cognitions and mood, and arousal criteria symptoms, in line with literature confirming higher vulnerability in women to trauma-related disorders [54]. It is also interesting that no statistically significant difference emerged in avoidance symptoms, highlighting the specific framework of the traumatic impact of a severe illness affecting a loved one. Higher trauma-related symptomatology among mothers could also be discussed in light of the frequent higher involvement in parenting duties [55]. In this regard, Yagmur Ozturk [56] and colleagues pointed out that mothers of autistic children are regularly engaged with them than fathers, heightening exposure to the child’s demandingness and neediness for care [57]. This also may render mothers at higher risk of difficulties in role balance (meaning they struggle to adequately distribute their time between parental responsibility and other aspects of life, such as work, social life, and hobbies) [56].

### 4.2. Role of Autistic Traits

The present study also investigated clinical correlates of ATs among parents of autistic children on the severity of the potentially distressing and traumatic impact of the first diagnosis. Our results were in line with recent findings suggesting a specific association of autism subthreshold spectrum symptoms with post-traumatic stress reactions [45]. Moreover, we found that increasing scoring on the AdAS-SR was associated with the presence of PTSD. There is evidence that, given the socio-cognitive characteristics of autism, such as mental rigidity, differences in information processing/perception, impaired emotional insight, and decreased goal-directedness, the application of cognitive coping and other adaptive strategies needed to face a traumatic event can be reduced, leading individuals with significant AT to exaggerated trauma responses [36]. In addition, impairments in cognitive flexibility and a difficulty in shifting focus may lead to increased rumination, which seems to be a nuclear element of the autistic spectrum symptomatology and that contributes to chronic traumatization that could impair individuals’ wellbeing [58,59]. These results added to previous data, which reported high levels of significant AT in different kinds of mental disorders, as well as a higher frequency of psychiatric conditions in parents of autistic subjects [44,60,61,62].

There are some possible links between trauma and autism. A previous study that found that people with autism, followed for one year after exposure to the 2009 L’Aquila earthquake, showed significantly lower adaptation than healthy controls [63]. One way autism may act as a susceptibility marker for PTSD is by increasing the likelihood of being exposed to traumatic situations. Stressful incidents such as bullying, teasing, and exclusion have been found to be particularly common among autistic people. In this context, we may hypothesize autistic parents could re-experience their childhood traumatic events through the experience of their child [31]. Both autistic children and adults may find it challenging to acquire socially acceptable habits. As a result, they may become more vulnerable to victimization [64]. Further, both conditions appear to share common issues with controlling emotions. People with PTSD frequently struggle to control their unpleasant emotions. Similarly, autistic subjects often show poorly differentiated emotional responses and self-knowledge and are more difficult to calm down when agitated [65].

### 4.3. Impact on Functioning

Prior studies have established the correlation between BAP in parents of recently diagnosed autistic children and poorer global functioning, particularly within the nonverbal communication AdAS domain [40]. Indeed, some typical autistic traits, like disruption in emotional processing [66] or cognitive rigidity, could impede the expression of adaptive capabilities needed to address the heightened caregiving demands of an autistic child, the acceptance of life prospect variations that come with a diagnosis of autism in one’s child, and the increased marital stress [67]. Moreover, individuals with significant ATs may find themselves lacking the social support networks that are considered a critical protective factor of health outcomes among families of children with a diagnosis of ASD [68,69]. Nevertheless, our study reveals that a more considerable impact on functioning arises from post-traumatic stress symptoms. Specifically, our regression analysis determined that a higher score on the TALS-SR predicted poorer social and occupational functioning measured by the SOFAS score. Therefore, while the presence of significant autistic traits predicted a higher intensity of post-traumatic stress spectrum symptomatology, it was this latter that had a more pronounced impact on overall functioning. This outcome is consistent with earlier research findings that show how partial or subthreshold PTSD symptoms are linked to difficulties adjusting to family, work and social situations as fully develop syndromes, both during the acute phase but also for some time after the traumatic exposure, particularly in mothers, who are most likely to develop PTSD after a traumatic experience regarding one’s child [70,71]. In the context of parents with autistic children, certain PTSD symptoms may directly impact the parents’ functioning. For instance, manifestations of hypervigilance could prompt frequent and inappropriate medical consultations, while avoidance behaviors resulting from trauma may establish a pattern of avoiding subsequent interactions with healthcare professionals. This pattern, in turn, poses challenges to the effectiveness of therapeutic interventions for both parents and their children [71,72].

### 4.4. Study Strengths and Limitations

Interpretation of our results should keep in mind some important limitations of the study. First, although the sample can be considered remarkable if we consider that parents were paired for one target child, a larger sample size would be warranted to corroborate the generalizability of our results. Second, the use of self-report instruments, despite a structured clinical interview to detect PTSD symptoms, may have given rise to feelings of shame for respondents expressing their symptoms. However, the use of spectrum assessments may allow us to capture more detailed pictures of the clinical symptoms. Third, we did not collect any data about who is the main caregiver of the child or regarding the presence of any co-occurring behavioral difficulties or other conditions. Fourth, we are aware of the fact that other factors non evaluated in the current investigation may contribute to PTSD reactions in parents of autistic children, including parental history of mental illness, other lifetime trauma events, social support, child autistic symptom severity, level of intervention received by children with autism, and sociodemographic characteristics (e.g., financial stress, multiple children in the household, multiple children with neurodevelopmental conditions) [73,74]. Further studies exploring these variables are therefore required to deepen the current understanding of risk and protective factors of parental PTSD in autistic children.

## 5. Conclusions

Our results shed some light on the possible psychopathological impact on caregivers of a lifelong diagnosis in one’s child, in this case, of autism, on parents. Despite the limitations mentioned above, this study offers the first contribution to the detection of post-traumatic stress symptoms based on DSM-5-TR criteria among parents of autistic children. Further, our results highlight the relevance of recognizing even the presence of significant AT in the mothers and fathers, as these seem to represent a risk factor for PTSD symptomatology. Considering the impact of autism among subjects primarily involved in the caregiving process of a child with a severe illness, the presence of post-traumatic stress symptoms could interfere with the sufferance and lack of efficacious support for the child and his/her treatment process. Due to some limitations of our study that are reported above, we cannot confidently generalize our results. More studies focused on this topic that take into consideration several variables (such as autistic symptom severity, parental history of mental illness, other life trauma events, assistance level needed by the child, or other relevant factors) are needed. These studies may help addressing targeted support for parents of autistic children in the early stage of the diagnosis of autism, with ad hoc dedicated services to improve their quality of life. Further, these studies may help to define specific intervention strategies [75] that may include structured psychoeducation programs aimed at enhancing parents’ self-efficacy and caregiving skills, together with psychological support to address trauma-related symptoms and avoid maladaptive coping strategies [30,36,72,76]. Moreover, acknowledging the possible presence of subthreshold autistic traits in some parents may be crucial to adapt interventions to the unique experience of parents on the autism spectrum [77].

## Figures and Tables

**Table 1:** Mean TALS domains scores and endorsement of DSM-5-TR PTSD criteria in the total sample and comparison between mothers and fathers.

	Total Sample Mean (±SD)	Father Mean (±SD)	Mother Mean (±SD)	z	*p*
**TALS-SR domain**					
Loss events (I)	3.70 (±1.79)	3.58 (±1.71)	4.50 (±1.89)	3.066	0.002
Grief reactions (II)	5.57 (±3.82)	6.06 (±4.55)	8.18 (±5.48)	3.024	0.002
Potentially traumatic events (III)	2.35 (±2.34)	2.84 (±2.85)	3.06 (±2.70)	0.746	0.456
Reactions to losses or upsetting events (IV)	3.91 (±3.08)	3.72 (±2.97)	5.58 (±3.58)	3.260	0.001
Re-experiencing (V)	1.45 (±1.70)	1.45 (±1.87)	2.50 (±2.42)	2.691	0.007
Avoidance and numbing (VI)	1.69 (±2.07)	1.70 (±2.11)	2.54 (±2.65)	1.690	0.090
Maladaptive coping (VII)	0.28 (±0.75)	0.22 (±0.71)	0.50 (±0.94)	2.145	0.032
Arousal (VIII)	0.55 (±1.03)	0.58 (±1.01)	1.04 (±1.37)	2.249	0.025
Total Symptomatologic score (IV + V + VI + VII + VIII)	9.91 (±8.42)	21.49 (±14.33)	29.37 (±17.23)	3.241	0.001
**PTSD**					
Criterion B	1.09 (±1.34)	0.79 (±1.10)	1.41 (±1.48)	2.500	0.011
Criterion C	0.60 (±0.80)	0.629 (±0.81)	0.60 (±0.80)	−0.293	0.769
Criterion D	1.33 (±1.60)	0.98 (±1.31)	1.69 (±1.80)	2.270	0.023
Criterion E	0.95 (±1.42)	0.64 (±1.11)	1.22 (±1.59)	2.640	0.008
**PTSD**					
Partial PTSD	49 (38.9%)	22 (34.9%)	27 (42.9%)	-	0.152 *
● 2 criteria	26 (20.6%)	13 (20.6%)	13 (20.6%)	-	1.000 *
● 3 criteria	23 (18.3%)	9 (14.3%)	14 (22.2%)	-	0.359 *
Full PTSD	13 (10.3%)	5 (7.9%)	8 (12.7%)	-	0.549 *

* McNemar Test was utilized to compare mothers and fathers on the rates of endorsement of diagnostic criteria.

**Table 2:** AQ and AdAS domains’ and total scores, and SOFAS total score in the total sample, and comparison between mothers and fathers.

	Total Sample Mean (±SD)	Fathers Mean (±SD)	Mothers Mean (±SD)	z	*p*
**AdAS Domain**					
Childhood/adolescence	4.58 (±3.75)	4.44 (±3.71)	4.72 (±3.82)	0.529	0.597
Verbal communication	3.24 (±2.78)	3.38 (±2.99)	3.11 (±2.57)	−0.294	0.769
Non-verbal communication	5.97 (±4.35)	5.79 (±4.72)	6.16 (±3.97)	0.894	0.371
Empathy	2.20 (±2.31)	2.33 (±2.39)	2.06 (±2.23)	−0.532	0.595
Inflexibility and adherence to routine	9.17 (±6.48)	8.73 (±6.65)	9.62 (±6.32)	1.026	0.305
Restricted interests and rumination	3.81 (±3.72)	3.53 (±3.66)	4.09 (±3.79)	0.815	0.415
Hyper– and hyporeactivity to sensory input	1.83 (±2.36)	1.68 (±2.41)	1.98 (±2.31)	0.966	0.334
**AQ domain**					
Social Skill	2.12 (±1.66)	1.95 (±1.61)	2.28 (±1.71)	1.044	0.296
Attention Switching	3.19 (±2.11)	3.10 (±2.13)	3.28 (±2.09)	0.513	0.608
Attention to detail	3.31 (±2.16)	3.43 (±2.53)	3.20 (±1.73)	0.236	0.813
Communication	1.64 (±1.74)	1.83 (±1.78)	1.45 (±1.69)	−1.522	0.128
Immagination	2.70 (±1.72)	3.10 (±1.86)	2.30 (±1.49)	−2.391	0.017
Total Score	13.02 (±5.98)	13.53 (±6.28)	12.52 (±5.67)	−0.910	0.363

**Table 3:** TALS-SR domain and total scores in parents with versus without significant AT (Independent-Samples Mann–Whitney U Test).

	Parents Without Significant AT (N = 96), Mean (±SD)	Parents with Significant AT (N = 28), Mean (±SD)	z	*p*
**TALS-SR domain**				
Loss events (I)	3.70 (±1.79)	5.21 (±1.57)	3.843	<0.001
Grief reactions (II)	5.57 (±3.82)	12.35 (±5.60)	5.614	<0.001
Potentially traumatic events (III)	2.35 (±2.34)	5.14 (±3.07)	4.670	<0.001
Reactions to losses or upsetting events (IV)	3.91 (±3.08)	7.07 (±3.42)	4.093	<0.001
Re-experiencing (V)	1.45 (±1.70)	3.7 (±2.85)	4.206	<0.001
Avoidance and numbing (VI)	1.69 (±2.07)	3.62 (±2.89)	3.398	<0.001
Maladaptive coping (VII)	0.28 (±0.75)	0.60 (±1.06)	1.464	0.143
Arousal (VIII)	0.55 (±1.03)	1.82 (±1.46)	4.696	<0.001
Personal characteristics/risk factors (IX)	1.07 (± 1.12)	2.42 (1.68)	4.045	<0.001
Total Symptomatologic score (IV + V + VI + VII + VIII)	7.91 (±6.91)	16.86 (±9.58)	4.577	<0.001
**SOFAS total score**	85.71 (±10.1)	75.37 (±12.18)	−4.132	<0.001

**Table 4:** (**A**) Logistic regression analysis for at least partial DSM-5-TR PTSD upon TALS-SR scoring as independent variable. (**B**) Linear regression analysis with SOFAS total score as independent variable.

**(A)**						
	**B (ES)**	**Odds** **Ratio**	***p***	**CI 95%**		
				**Upper**	**Lower**	
AdAS Total score	0.044 (0.012)	1.045	<0.001	3.677	1.021	
Gender	0.539 (0.390)	1.713	0.167	1.07	0.799	
k	−2.152 (0.713)	0.116	0.003	-	-	
**(B)**						
	**B (ES)**	**β**	***p***	**Semipartial** **Correlation**	**CI 95%**	
					**Upper**	**Lower**
ADAS total score	−0.111 (0.059)	−0.195	0.064	−0.152	0.007	−0.229
TALS-SR Total Symptomatologic score	−0.433 (0.149)	−0.313	0.004	−0.236	−0.138	−0.729
Gender	−2.091 (1.943)	−0.092	0.284	−0.087	1.759	−5.940
k	94.271 (3.287)		<0.001		100.782	87.760

(**A**) R = 0.155, R^2^ = 0.207; (**B**) R = 0.484, R^2^ = 0.214.

## Data Availability

The original contributions presented in the study are included in the article, further inquiries can be directed to the corresponding author/s upon reasonable request.

## References

[1] Dewan T., Birnie K., Drury J., Jordan I., Miller M., Neville A., Noel M., Randhawa A., Zadunayski A., Zwicker J. (2023). Experiences of Medical Traumatic Stress in Parents of Children with Medical Complexity. Child Care Health Dev..

[2] Barak-Levy Y., Paryente B. (2023). Diving into the Resolution Process: Parent’s Reactions to Child’s Diagnosis. Int. J. Environ. Res. Public Health.

[3] Feudtner C., Nye R.T., Boyden J.Y., Schwartz K.E., Korn E.R., Dewitt A.G., Waldman A.T., Schwartz L.A., Shen Y.A., Manocchia M. (2021). Association Between Children with Life-Threatening Conditions and Their Parents’ and Siblings’ Mental and Physical Health. JAMA Netw. Open.

[4] Nelson M., Kelly D., McAndrew R., Smith P. (2017). ‘Just Gripping My Heart and Squeezing’: Naming and Explaining the Emotional Experience of Receiving Bad News in the Paediatric Oncology Setting. Patient Educ. Counsel..

[5] Carmassi C., Dell’Oste V., Foghi C., Bertelloni C.A., Conti E., Calderoni S., Battini R., Dell’Osso L. (2020). Post-Traumatic Stress Reactions in Caregivers of Children and Adolescents/Young Adults with Severe Diseases: A Systematic Review of Risk and Protective Factors. Int. J. Environ. Res. Public Health.

[6] Lord B., Ungerer J., Wastell C. (2008). Implications of Resolving the Diagnosis of PKU for Parents and Children. J. Pediatr. Psychol..

[7] American Psychiatric Association (2022). Diagnostic and Statistical Manual of Mental Disorders: DSM-5-Tr.

[8] Dell’Osso L., Carmassi C., Rucci P., Conversano C., Shear M.K., Calugi S., Jack D., Endicott J., Fagiolini A., Cassano G.B. (2009). A Multidimensional Spectrum Approach to Post-Traumatic Stress Disorder: Comparison between the Structured Clinical Interview for Trauma and Loss Spectrum (SCI-TALS) and the Self-Report Instrument (TALS-SR). Compr. Psychiatry.

[9] McCarthy M.C., Ashley D.M., Lee K.J., Anderson V.A. (2012). Predictors of Acute and Posttraumatic Stress Symptoms in Parents Following Their Child’s Cancer Diagnosis. J. Trauma. Stress.

[10] Stuber M.L., Shemesh E. (2006). Post-Traumatic Stress Response to Life-Threatening Illnesses in Children and Their Parents. Child Adolesc. Psychiatr. Clin..

[11] Orsini A., Corsi M., Pedrinelli V., Santangelo A., Bertelloni C., Dell’oste V., Cordelli D., Perrone A., Parini L., Lanari M. (2021). Post-traumatic stress, anxiety, and depressive symptoms in caregivers of children tested for COVID-19 in the acute phase of the Italian outbreak. J. Psychiatr. Res..

[12] Carmassi C., Barberi F.M., Cordone A., Maglio A., Dell’Oste V., Dell’Osso L. (2020). Trauma, PTSD and post-traumatic stress spectrum: 15 years’ experience on a multidimensional approach to trauma related psychopathology. J. Psychopathol..

[13] Cernvall M., Alaie I., von Essen L. (2012). The Factor Structure of Traumatic Stress in Parents of Children with Cancer: A Longitudinal Analysis. J. Pediatr. Psychol..

[14] Carmassi C., Corsi M., Bertelloni C., Pedrinelli V., Massimetti G., Peroni D., Bonuccelli A., Orsini A., Dell’Osso L. (2019). Post-traumatic stress and major depressive disorders in parent caregivers of children with a chronic disorder. Psychiatry Res..

[15] Horsch A., McManus F., Kennedy P. (2012). Cognitive and Non-Cognitive Factors Associated with Posttraumatic Stress Symptoms in Mothers of Children with Type 1 Diabetes. Behav. Cogn. Psychother..

[16] Phipps S., Long A., Hudson M., Rai S.N. (2005). Symptoms of Post-Traumatic Stress in Children with Cancer and Their Parents: Effects of Informant and Time from Diagnosis. Pediatr. Blood Cancer.

[17] Dolgin M.J., Phipps S., Fairclough D.L., Sahler O.J.Z., Askins M., Noll R.B., Butler R.W., Varni J.W., Katz E.R. (2007). Trajectories of Adjustment in Mothers of Children with Newly Diagnosed Cancer: A Natural History Investigation. J. Pediatr. Psychol..

[18] Wilcoxon L.A., Meiser-Stedman R., Burgess A. (2021). Post-Traumatic Stress Disorder in Parents Following Their Child’s Single-Event Trauma: A Meta-Analysis of Prevalence Rates and Risk Factor Correlates. Clin. Child Fam. Psychol. Rev..

[19] Franck L.S., McQuillan A., Wray J., Grocott M.P.W., Goldman A. (2010). Parent Stress Levels during Children’s Hospital Recovery after Congenital Heart Surgery. Pediatr. Cardiol..

[20] Muscara F., Burke K., McCarthy M.C., Anderson V., Hearps S.J., Dimovski A., Nicholson J.M. (2015). Parent Distress Reactions Following a Serious Illness or Injury in Their Child: A Protocol Paper for the Take a Breath Cohort Study. BMC Psychiatry.

[21] Carmassi C., Corsi M., Bertelloni C.A., Pedrinelli V., Massimetti G., Peroni D., Bonuccelli A., Orsini A., Dell’osso L. (2020). Post-Traumatic Stress Spectrum Symptoms in Parents of Children Affected by Epilepsy: Gender Differences. Seizure.

[22] Bernstein-Eliav M., Tavor I. (2024). The Prediction of Brain Activity from Connectivity: Advances and Applications. Neuroscientist.

[23] Wang M., Xu D., Zhang L., Jiang H. (2023). Application of Multimodal MRI in the Early Diagnosis of Autism Spectrum Disorders: A Review. Diagnostics.

[24] Pagnozzi A.M., Conti E., Calderoni S., Fripp J., Rose S.E. (2018). A Systematic Review of Structural MRI Biomarkers in Autism Spectrum Disorder: A Machine Learning Perspective. Int. J. Dev. Neurosci..

[25] Billeci L., Calderoni S., Conti E., Gesi C., Carmassi C., Dell’Osso L. (2016). The Broad Autism (Endo)Phenotype: Neurostructural and Neurofunctional Correlates in Parents of Individuals with Autism Spectrum Disorders. Front. Neurosci..

[26] Serrano L., Vela E., Martín L. (2023). Analysis of the Functioning of Families of Children with Autism Spectrum Disorder: A Psychometric Study of the Family APGAR Scale. Int. J. Environ. Res. Public Health.

[27] Firth I., Dryer R. (2013). The Predictors of Distress in Parents of Children with Autism Spectrum Disorder. J. Intellect. Dev. Disabil..

[28] Benson P.R. (2006). The Impact of Child Symptom Severity on Depressed Mood Among Parents of Children with ASD: The Mediating Role of Stress Proliferation. J. Autism Dev. Disord..

[29] Wade J.L., Cox N.B., Reeve R.E., Hull M. (2014). Brief Report: Impact of Child Problem Behaviors and Parental Broad Autism Phenotype Traits on Substance Use among Parents of Children with ASD. J. Autism Dev. Disord..

[30] Lai W.W., Goh T.J., Oei T.P.S., Sung M. (2015). Coping and Well-Being in Parents of Children with Autism Spectrum Disorders (ASD). J. Autism Dev. Disord..

[31] Smit S., Hopper J. (2023). Love, Joy, and a Lens of Childhood Trauma: Exploring Factors That Impact the Mental Health and Well-Being of Autistic Parents via Iterative Phenomenological Analysis. Autism Adulthood.

[32] Bitsika V., Sharpley C.F., Bell R. (2013). The Buffering Effect of Resilience upon Stress, Anxiety and Depression in Parents of a Child with an Autism Spectrum Disorder. J. Dev. Phys. Disabil..

[33] Davis N., Carter A. (2008). Parenting Stress in Mothers and Fathers of Toddlers with Autism Spectrum Disorders: Associations with Child Characteristics. J. Autism Dev. Disord..

[34] Osborne L., Mchugh L., Saunders J., Reed P. (2008). A Possible Contra-Indication for Early Diagnosis of Autistic Spectrum Conditions: Impact on Parenting Stress. Res. Autism Spectr. Disord..

[35] Goin-Kochel R.P., Mackintosh V.H., Myers B.J. (2006). How Many Doctors Does It Take to Make an Autism Spectrum Diagnosis?. Autism.

[36] Kerns C.M., Newschaffer C.J., Berkowitz S.J. (2015). Traumatic Childhood Events and Autism Spectrum Disorder. J. Autism Dev. Disord..

[37] Rumball F., Happé F., Grey N. (2020). Experience of Trauma and PTSD Symptoms in Autistic Adults: Risk of PTSD Development Following DSM-5 and Non-DSM-5 Traumatic Life Events. Autism Res..

[38] Aguiar M.C.M.D., Pondé M.P. (2020). Autism: Impact of the Diagnosis in the Parents. J. Bras. Psiquiatr..

[39] Dell’Osso L., Dalle Luche R., Gesi C., Moroni I., Carmassi C., Maj M. (2016). From Asperger’s Autistischen Psychopathen to DSM-5 Autism Spectrum Disorder and Beyond: A Subthreshold Autism Spectrum Model. Clin. Pract. Epidemiol. Ment. Health.

[40] Carpita B., Carmassi C., Calderoni S., Muti D., Muscarella A., Massimetti G., Cremone I.M., Gesi C., Conti E., Muratori F. (2020). The Broad Autism Phenotype in Real-Life: Clinical and Functional Correlates of Autism Spectrum Symptoms and Rumination among Parents of Patients with Autism Spectrum Disorder. CNS Spectr..

[41] Ingersoll B., Hambrick D.Z. (2011). The Relationship between the Broader Autism Phenotype, Child Severity, and Stress and Depression in Parents of Children with Autism Spectrum Disorders. Res. Autism Spectr. Disord..

[42] Marriott E., Stacey J., Hewitt O.M., Verkuijl N.E. (2022). Parenting an Autistic Child: Experiences of Parents with Significant Autistic Traits. J. Autism Dev. Disord..

[43] Camm-Crosbie L., Bradley L., Shaw R., Baron-Cohen S., Cassidy S. (2019). “People like Me Don’t Get Support”: Autistic Adults’ Experiences of Support and Treatment for Mental Health Difficulties, Self-Injury and Suicidality. Autism.

[44] Carmassi C., Corsi M., Bertelloni C.A., Carpita B., Gesi C., Pedrinelli V., Massimetti G., Peroni D.G., Bonuccelli A., Orsini A. (2018). Mothers and Fathers of Children with Epilepsy: Gender Differences in Post-Traumatic Stress Symptoms and Correlations with Mood Spectrum Symptoms. Neuropsychiatr. Dis. Treat..

[45] Dell’Osso L., Corsi M., Gesi C., Bertelloni C., Massimetti G., Peroni D., Bonuccelli A., Orsini A., Carmassi C. (2018). Adult Autism Subthreshold Spectrum (AdAS Spectrum) in Parents of Pediatric Patients with Epilepsy: Correlations with Post-Traumatic Stress Symptoms. Compr. Psychiatry.

[46] McDonnell C.G., Delucia E.A. (2021). Pregnancy and Parenthood Among Autistic Adults: Implications for Advancing Maternal Health and Parental Well-Being. Autism Adulthood.

[47] Baron-Cohen S., Wheelwright S., Skinner R., Martin J., Clubley E. (2001). The Autism-Spectrum Quotient (AQ): Evidence from Asperger Syndrome/High-Functioning Autism, Males and Females, Scientists and Mathematicians. J. Autism Dev. Disord..

[48] Wheelwright S., Auyeung B., Allison C., Baron-Cohen S. (2010). Defining the Broader, Medium and Narrow Autism Phenotype among Parents Using the Autism Spectrum Quotient (AQ). Mol. Autism.

[49] McLaughlin K.A., Koenen K.C., Friedman M.J., Ruscio A.M., Karam E.G., Shahly V., Stein D.J., Hill E.D., Petukhova M., Alonso J. (2015). Subthreshold Posttraumatic Stress Disorder in the World Health Organization World Mental Health Surveys. Biol. Psychiatry.

[50] Goldman H.H., Skodol A.E., Lave T.R. (1992). Revising Axis V for DSM-IV: A Review of Measures of Social Functioning. Am. J. Psychiatry.

[51] Cabizuca M., Marques-Portella C., Mendlowicz M.V., Coutinho E.S.F., Figueira I. (2009). Posttraumatic Stress Disorder in Parents of Children with Chronic Illnesses: A Meta-Analysis. Health Psychol..

[52] Olff M. (2017). Sex and Gender Differences in Post-Traumatic Stress Disorder: An Update. Eur. J. Psychotraumatology.

[53] Le Gouëz M., Alvarez L., Rousseau V., Hubert P., Abadie V., Lapillonne A., Kermorvant-Duchemin E., Simeoni U. (2016). Posttraumatic Stress Reactions in Parents of Children Esophageal Atresia. PLoS ONE.

[54] Kessler R.C., Wai T.C., Demler O., Walters E.E. (2005). Prevalence, Severity, and Comorbidity of 12-Month DSM-IV Disorders in the National Comorbidity Survey Replication. Arch. Gen. Psychiatry.

[55] Bornstein M.H., Sawyer J. (2008). Family Systems. Blackwell Handbook of Early Childhood Development.

[56] Ozturk Y., Riccadonna S., Venuti P. (2014). Parenting Dimensions in Mothers and Fathers of Children with Autism Spectrum Disorders. Res. Autism Spectr. Disord..

[57] Keller D., Honig A.S. (2004). Maternal and Paternal Stress in Families with School-Aged Children with Disabilities. Am. J. Orthopsychiatry.

[58] King R. (2010). Complex Post-Traumatic Stress Disorder: Implications for Individuals with Autism Spectrum Disorders-Part I. J. Dev. Disabil..

[59] Williams Z.J., McKenney E.E., Gotham K.O. (2021). Investigating the Structure of Trait Rumination in Autistic Adults: A Network Analysis. Autism.

[60] Yin W., Pulakka A., Reichenberg A., Kolevzon A., Ludvigsson J.F., Risnes K., Lahti-Pulkkinen M., Persson M., Silverman M.E., Åden U. (2024). Association between Parental Psychiatric Disorders and Risk of Offspring Autism Spectrum Disorder: A Swedish and Finnish Population-Based Cohort Study. Lancet Reg. Health—Eur..

[61] Schnabel A., Youssef G.J., Hallford D.J., Hartley E.J., McGillivray J., Stewart M., Forbes D., Austin D.W. (2020). Psychopathology in Parents of Children with Autism Spectrum Disorder: A Systematic Review and Meta-Analysis of Prevalence. Autism.

[62] Dell’Osso L., Carpita B., Cremone I.M., Muti D., Diadema E., Barberi F.M., Massimetti G., Brondino N., Petrosino B., Politi P. (2019). The mediating effect of trauma and stressor related symptoms and ruminations on the relationship between autistic traits and mood spectrum. Psychiatry Res..

[63] Valenti M., Ciprietti T., Egidio C.D., Gabrielli M., Masedu F., Tomassini A.R. (2012). Adaptive Response of Children and Adolescents with Autism to the 2009 Earthquake in L’Aquila, Italy. J. Autism Dev. Disord..

[64] Takara K., Kondo T. (2014). Autism Spectrum Disorder among First-Visit Depressed Adult Patients: Diagnostic Clues from Backgrounds and Past History. Gen. Hosp. Psychiatry.

[65] Mazefsky C.A., White S.W. (2014). Emotion Regulation: Concepts and Practice in Autism Spectrum Disorder. Child Adolesc. Psychiatr. Clin. N. Am..

[66] Desquenne Godfrey G., Downes N., Cappe E. (2024). A Systematic Review of Family Functioning in Families of Children on the Autism Spectrum. J. Autism Dev. Disord..

[67] Hartley S.L., Barker E.T., Seltzer M.M., Floyd F., Greenberg J., Orsmond G., Bolt D. (2010). The relative risk and timing of divorce in families of children with an autism spectrum disorder. J. Fam. Psychol..

[68] Feng Y., Zhou X., Qin X., Cai G., Lin Y., Pang Y., Chen B., Deng T., Zhang L. (2022). Parental Self-Efficacy and Family Quality of Life in Parents of Children with Autism Spectrum Disorder in China: The Possible Mediating Role of Social Support. J. Pediatr. Nurs..

[69] Estell D.B., Farmer T.W., Irvin M.J., Crowther A., Akos P., Boudah D.J. (2009). Students with Exceptionalities and the Peer Group Context of Bullying and Victimization in Late Elementary School. J. Child Fam. Stud..

[70] Tolin D.F., Foa E.B. (2006). Sex Differences in Trauma and Posttraumatic Stress Disorder: A Quantitative Review of 25 Years of Research. Psychol. Bull..

[71] Stuber M.L., Christakis D.A., Houskamp B., Kazak A.E. (1996). Posttrauma Symptoms in Childhood Leukemia Survivors and Their Parents. Psychosomatics.

[72] Pelcovitz D., Libov B.G., Mandel F., Kaplan S., Weinblatt M., Septimus A. (1998). Posttraumatic Stress Disorder and Family Functioning in Adolescent Cancer. J. Trauma. Stress.

[73] Xiong T., McGrath P.J., Stewart S.H., Bagnell A., Kaltenbach E. (2022). Risk and Protective Factors for Posttraumatic Stress and Posttraumatic Growth in Parents of Children with Intellectual and Developmental Disorders. Eur. J. Psychotraumatology.

[74] Xu L., Song L., Xiong Z., Chen J. (2024). The Relationship between Perceived Social Support and Rumination among Parents of Children with Autism: Moderating Effect of the Degree of Intervention Received by Children. Front. Psychiatry.

[75] Dai Y., Chen M., Deng T., Huang B., Ji Y., Feng Y., Liu S., Zhang L. (2024). The Importance of Parenting Self-Efficacy and Social Support for Family Quality of Life in Children Newly Diagnosed with Autism Spectrum Disorder: A One-Year Follow-up Study. Autism Res..

[76] Lehman L.L., Maletsky K., Beaute J. (2020). Prevalence of Symptoms of Anxiety, Depression, and Post-Traumatic Stress Disorder in Parents and Children Following Pediatric Stroke. J. Child Neurol..

[77] Lockington D.C., Gullon-Scott F. (2025). The Lived Experiences of Autistic Mothers: A Systematic Review and Thematic Synthesis of Qualitative Evidence. Autism Dev. Lang. Impair..

